# Supplementary material for: Osteopathy students profile in Italy: A cross sectional census
Source: PLoS One. 2021 Feb 24;16(2):e0247405. doi: 10.1371/journal.pone.0247405 (PMC7904159; doi:10.1371/journal.pone.0247405)
Supplement: S1 File — (DOCX) [file pone.0247405.s001.docx]

Questionnaire

OSA - OSTEOPATHY STUDENTS ANALYSIS: OSTEOPATHY STUDENTS PROFILE IN ITALY

The aim of this survey is to describe the profile of Osteopathy students in Italy.

The present study considers the socio-demographic characteristics (11 items), the geographical distribution (5 items), the health status (3 items), and previous, ongoing education specifications (16 items).

Reading and fulfilling will require approximately 3 minutes. For each item/question you need to cross only a single square.

**1. Section: Socio-demographic characteristics**

1. Age

- 18
- 19
- 20
- 21
- 22
- 23
- 24
- 25
- 26
- 27
- 28
- 29
- 30
- 31
- 32
- 33
- 34
- 35
- 36
- 37
- 38
- 39
- 40
- 41
- 42
- 43
- 44
- 45
- 46
- 47
- 48
- 49
- 50
- 51
- 52
- 53
- 54
- 55
- 56
- 57
- 58
- 59
- 60
- 61
- 62
- 63
- 64
- 65
- 66
- 67
- 68
- 69
- 70

2. Gender

- Woman
- Man
- Other

3. Ethnic group

- European
- Arab
- Central Asian
- Indian
- Malagasy
- Indian-Black American
- Mixed
- Black
- East Asian
- Inuit
- Amerindian
- Mestizo
- American Multiracial
- Australian Multiracial

4. Religious orientation

- Atheist
- Christian
- Hebrew
- Islamic
- Other

5. Which of the following working conditions are you currently in?

- None occupation
- Intermittent or occasional work
- Fixed term employee work
- Permanent employee work
- Self -employed

6. Indicate your mother’s highest level of education:

- Primary school
- Secondary school
- High School
- Bachelor or Master Degree
- PhD

7. Indicate your father’s highest level of education:

- Primary school
- Secondary school
- High School
- Bachelor or Master Degree
- PhD

8. Indicate which profession your mother is holding or has pursued:

- Self- employment
- Employee (fixed-term contract)
- Employee (permanent contract)
- Housewife

9. Indicate which profession your father is holding or has pursued:

- Self- employment
- Employee (fixed-term contract)
- Employee (permanent contract)
- Housewife

10. State your original family income:

- 0 - 15.000 €/year
- 15.001 e 28.000 €/year
- 28.001 e 55.000 €/year
- 55.001 e 75.000 €/year
- Over 75.000 €/year
- I don’t know

11. Favourite hobbies during your free time:

- Outdoor activities
- Art
- Cinema
- Cooking
- Photography
- Internet
- Reading
- Music
- Shopping
- Social life
- Sport
- Theatre
- Watching TV
- Travel
- Video Games
- Other

**2. Section: Geographical distribution**

12. Country of birth:

- Afghanistan
- Albania
- Algeria
- Andorra
- Angola
- Antigua e Barbuda
- Argentina
- Armenia
- Austria
- Australia
- Brasil
- Bolivia
- Bosnia and Herzegovina
- Botswana
- Brunei
- Bulgaria
- Burkina Faso
- Burundi
- Cambodia
- Cameroon
- Canada
- Cape Verde
- Chad
- Chile
- China
- Cipro
- Colombia
- Comoro Islands
- Costa Rica
- Croatia
- Cuba
- Czech Republic
- Denmark
- Dominica
- Ecuador
- Egypt
- El Salvador
- Equatorial Guinea
- Eritrea
- Estonia
- Ethiopia
- Fiji
- Finland
- France
- Gabon
- Gambia
- Georgia
- Germany
- Ghana
- Jamaica
- Japan
- Jordan
- Gibuti
- Greece
- Grenada
- Guatemala
- Guinea
- Guinea-Bissau
- Guyana
- Haiti
- Honduras
- Hungary
- India
- Indonesia
- Iran
- Iraq
- Ireland
- Island
- Ivory Coast
- Israel
- Italia
- Kazakhstan
- Kenya
- Kirghizstan
- Kiribati
- Kuwait
- Laos
- Latvia
- Lebanon
- Lesotho
- Liberia
- Libya
- Liechtenstein
- Lithuania
- Luxembourg
- Macedonia
- Madagascar
- Malawi
- Maldives
- Malesia
- Mali
- Malta
- Marshall Island
- Mauritania
- Mauritius
- Mexico
- Micronesia
- Moldavia
- Monaco
- Mongolia
- Montenegro
- Morocco
- Mozambique
- Myanmar
- Namibia
- Nauru
- Nephal
- Netherlands
- New Zealand
- Nicaragua
- Niger
- Nigeria
- Norway
- North Korea
- Oman
- Pakistan
- Palau
- Palestine
- Panama
- Papua New Guinea
- Paraguay
- Peru
- Philippines
- Polonia
- Portugal
- Qatar
- Russia
- Solomon Islands
- Saudi Arabia
- South-Africa
- Sudan
- South Korea
- South Sudan
- Suriname
- Sweden
- Switzerland
- Swaziland
- Tajikistan
- Taiwan
- Tanzania
- Thailand
- Timor Est
- Togo
- Tonga
- Trinidad e Tobago
- Tunisia
- Turkey
- Turkmenistan
- Tuvalu
- Ukraine
- Uganda
- United Arab Emirates
- United Kingdom
- United States of America
- Uruguay
- Uzbekistan
- Vanuatu
- Vatican City State
- Venezuela
- Vietnam
- Yemen
- Zambia
- Zimbabwe

13. Region of birth:

- Abruzzo
- Valle d’Aosta
- Basilicata
- Calabria
- Campania
- Emilia-Romagna
- Friuli-Venezia Giulia
- Lazio
- Liguria
- Lombardia
- Marche
- Molise
- Piemonte
- Puglia
- Sardegna
- Sicilia
- Toscana
- Trentino-Alto Adige
- Umbria
- Veneto
- Foreign

14. Region of residence:

- Abruzzo
- Valle d’Aosta
- Basilicata
- Calabria
- Campania
- Emilia-Romagna
- Friuli-Venezia Giulia
- Lazio
- Liguria
- Lombardia
- Marche
- Molise
- Piemonte
- Puglia
- Sardegna
- Sicilia
- Toscana
- Trentino-Alto Adige
- Umbria
- Veneto
- Foreign

15. Region where you study:

- Abruzzo
- Valle d’Aosta
- Basilicata
- Calabria
- Campania
- Emilia-Romagna
- Friuli-Venezia Giulia
- Lazio
- Liguria
- Lombardia
- Marche
- Molise
- Piemonte
- Puglia
- Sardegna
- Sicilia
- Toscana
- Trentino-Alto Adige
- Umbria
- Veneto
- Foreign

16. Are you an on-site or off-site student (from your place of residence)?

- On-site
- Off-site

**3. Section: State of health**

17. Do you think your state of health is?

very bad 1 2 3 4 5 really good

18. Are you suffering from a condition that relates to?

- Central nervous system
- Peripheral nervous system
- Sensorial system
- Musculoskeletal system
- Cardio-circulatory system
- Endocrine system
- Respiratory system
- None
- Other

19. Are you entitled to an invalidity pension?

- Yes
- no

**4. Section: Education**

20. Why did you choose Osteopathy course?

- Interest in the subjects taught
- Desire to prepare myself for a useful profession for others
- Continuity with the training course carried out
- Good chances of finding a job
- Non-admission to other courses
- Economic prospects of the profession
- Other

21. Your choice was:

- An autonomous decision
- A suggestion of the teachers met in the previous training courses
- A suggestion from family/friends
- A suggestion from guidance centres
- A suggestion from the mass media (TV, radio, internet)
- It was a random choice
- Determined by direct experience as an osteopath patient
- Other

22. What kind of programme did you choose?

- T1 (full-time)
- T2 (part-time)

23. Which school are you enrolled in?

- ABEOS - Scuola di Osteopatia (L’Aquila)
- ABEOS - Scuola di Osteopatia (Bologna)
- ABEOS - Scuola di Osteopatia (Raiano - AQ)
- AEMO - Accademia Europea di Medicina Osteopatica (Aversa - CE)
- AIFROMM - Accademia Internazionale di Formazione e Ricerca in Osteopata e Medicina Manuale (Bergamo)
- AIMO - Accademia Italiana Medicina Osteopatica (Saronno - VA)
- AIOT - Accademia Italiana Osteopatia Tradizionale ABRUZZO (Pescara)
- AIOT - Accademia Italiana Osteopatia Tradizionale MARCHE (Civitanova Marche - MC)
- ATSAI - A.T. Still Academy Italia (Bari)
- CERDO - Centre pour l’Étude, la Recherche et la Diffusion Ostéopathiques (Roma)
- CIO - Collegio Italiano di Osteopatia (Parma)
- CHINESIS IFOP - Istituto di Formazione in Osteopatia e in Posturologia (Roma)
- CSDOI - Centro Studi di Osteopatia Italiano (Catania)
- CSOT - Centro Studi di Osteopatia Tradizionale (Roma)
- FULCRO - Istituto Osteopatico Fulcro (Treviso)
- IAOM AISERCO - International Academy of Osteopathic Medicine (Palermo)
- ICOMM - International College of Osteopathic Manual Medicine (Roma)
- ISO/IIO - Istituto Superiore Osteopatia (Milano)
- OSCE - Osteopathic Spine Center Education (Bologna)
- OSTEOPATHIC COLLEGE (Trieste)
- SOFI - Scuola di Osteopatia Franco Italiana (Pisa)

24. Why did you apply in the chosen school?

- School Prestige
- The quality of teaching and teaching staff
- The most convenient to reach
- Advantageous registration fees
- Release of foreign university degree
- Other

25. Type of high school attended:

- Arts studies
- Classical studies
- Linguistic studies
- Scientific studies
- Pedagogical studies
- Technical studies
- Other

26. Year of attainment of the high school diploma:

- Before December 1997
- After December 1997

27. Have you ever failed a class at high school?

- Yes
- no

28. High school graduation mark:

- 36
- 37
- 38
- 39
- 40
- 41
- 42
- 43
- 44
- 45
- 46
- 47
- 48
- 49
- 50
- 51
- 52
- 53
- 54
- 55
- 56
- 57
- 58
- 59
- 60
- 61
- 62
- 63
- 64
- 65
- 66
- 67
- 68
- 69
- 70
- 71
- 72
- 73
- 74
- 75
- 76
- 77
- 78
- 79
- 80
- 81
- 82
- 83
- 84
- 85
- 86
- 87
- 88
- 89
- 90
- 91
- 92
- 93
- 94
- 95
- 96
- 97
- 98
- 99
- 100

29. Academic title:

- Bachelor (BSc)
- Master's (MSc)
- PhD (PhD)
- None

30. Attempts of gain access to a degree course:

- 0
- 1
- 2
- 3
- 4
- 5

31. Degree score (Highest academic level reached):

- None
- 66
- 67
- 68
- 69
- 70
- 71
- 72
- 73
- 74
- 75
- 76
- 77
- 78
- 79
- 80
- 81
- 82
- 83
- 84
- 85
- 86
- 87
- 88
- 89
- 90
- 91
- 92
- 93
- 94
- 95
- 96
- 97
- 98
- 99
- 100
- 101
- 102
- 103
- 104
- 105
- 106
- 107
- 108
- 109
- 110
- 110 with lode

32. Number of osteopathy schools attended:

- 1
- 2
- 3

33.Which year of course are you attending in your current school of osteopathy?

- 1
- 2
- 3
- 4
- 5
- 6

34. Have you ever missed a school year or had to repeat during your osteopathy course?

- Yes
- no

35. Do you intend to practice the profession in your residence region or elsewhere after your diploma?

- Residence region
- Somewhere else
