# Supplementary material for: Osteopathy students profile in Italy: A cross sectional census
Source: PLoS One. 2021 Feb 24;16(2):e0247405. doi: 10.1371/journal.pone.0247405 (PMC7904159; doi:10.1371/journal.pone.0247405)
Supplement: S1 Table — (DOCX) [file pone.0247405.s003.docx]

| **Working conditions** | | |
| --- | --- | --- |
| **Parameter** | **N** | **%** |
| None occupation | 907 | 24.1 |
| Intermittent or occasional work | 737 | 19.6 |
| Fixed term employee work | 471 | 12.5 |
| Permanent employee work | 324 | 8.6 |
| Self-employed | 1323 | 35.2 |
| Question 5. Working conditions |  |  |

| **Favourite hobbies** | | |
| --- | --- | --- |
| **Parameter** | **N** | **%** |
| Outdoor activities | 1861 | 49.5 |
| Art | 646 | 17.2 |
| Cinema | 1175 | 31.2 |
| Cooking | 952 | 25.3 |
| Photography | 566 | 15.0 |
| Internet | 863 | 22.9 |
| Reading | 1250 | 33.2 |
| Music | 1755 | 46.7 |
| Shopping | 744 | 19.8 |
| Social life | 2066 | 54.9 |
| Sport | 2868 | 76.2 |
| Theatre | 317 | 8.4 |
| Watching TV | 556 | 14.,8 |
| Travel | 2068 | 55.0 |
| Video Games | 444 | 11.,8 |
| Altro | 311 | 8.3 |
| Question 11. Favourite hobbies |  |  |

| **Country of birth** | | |
| --- | --- | --- |
| **Parameter** | **N** | **%** |
| Albania | 14 | 0.37 |
| Argentina | 1 | 0.03 |
| Austria | 1 | 0.03 |
| Bolivia | 3 | 0.08 |
| Bosnia & Erzegovina | 2 | 0.05 |
| Brasil | 5 | 0.13 |
| Canada | 1 | 0.03 |
| Capo Verde | 1 | 0.03 |
| Chile | 1 | 0.03 |
| Cina | 1 | 0.03 |
| Colombia | 1 | 0.03 |
| Croatia | 1 | 0.03 |
| Ecuador | 3 | 0.08 |
| Egypt | 1 | 0.03 |
| Ethiopia | 3 | 0.08 |
| France | 7 | 0.19 |
| Germany | 6 | 0.16 |
| Greece | 4 | 0,11 |
| India | 3 | 0.08 |
| Iran | 3 | 0.08 |
| Ireland | 1 | 0.03 |
| Italy | 3657 | 97.21 |
| Kazakistan | 1 | 0.03 |
| Kenya | 1 | 0.03 |
| Latvia | 1 | 0.03 |
| Lebanon | 1 | 0.03 |
| Mexico | 1 | 0.03 |
| Moldavia | 2 | 0.05 |
| Netherlands | 1 | 0.03 |
| Peru | 1 | 0.03 |
| Polonia | 2 | 0.05 |
| Romania | 10 | 0.27 |
| Russia | 2 | 0.05 |
| S.Marino | 4 | 0.11 |
| Spain | 1 | 0.03 |
| Switzerland | 5 | 0.13 |
| Tagikistan | 1 | 0.03 |
| Ukraine | 4 | 0.11 |
| United States of America | 2 | 0.05 |
| Venezuela | 1 | 0.03 |
| Vietnam | 1 | 0.03 |
| Question 12. Country of birth |  |  |

| **Site of study** | | |
| --- | --- | --- |
| **Parameter** | **N** | **%** |
| Offsite | 1500 | 39.9 |
| Onsite | 2262 | 60.1 |
| Question 16. Site of study |  |  |

| **Invalid pension** | | |
| --- | --- | --- |
| **Parameter** | **N** | **%** |
| No | 3748 | 99.6 |
| Yes | 14 | 0.4 |
| Question 19. Site of study |  |  |

| **Reason for choosing Osteopathy** | | |
| --- | --- | --- |
| **Parameter** | **N** | **%** |
| Interest in the subject taught | 2278 | 60,6 |
| Deside to prepare myself for a useful profession for other | 2376 | 63,2 |
| Continuity with the training course carried out | 1243 | 33,0 |
| Non-admission to other courses | 296 | 7,9 |
| Economic prospects of the profession | 895 | 23,8 |
| Other | 282 | 7,5 |
| Question 20. Reason for choosing Osteopathy |  |  |

| **Choice to engage in the osteopathic school** | | |
| --- | --- | --- |
| **Parameter** | **N** | **%** |
| An autonomous decision | 3111 | 82.7 |
| A suggestion of the teacher met in the previous training courses | 209 | 5.6 |
| A suggestion from family/friends | 746 | 19.8 |
| A suggestion from guidance centres | 56 | 1.5 |
| A suggestion from the mass media (TV, radio, Internet) | 22 | 0.6 |
| It was a random choice | 85 | 2.3 |
| Determined by direct experience as an osteopath patient | 911 | 24.2 |
| Other | 85 | 2.3 |
| Question 21. Choice to engage in the osteopathic school |  |  |

| **Reason to apply in the chosen school** | | |
| --- | --- | --- |
| **Parameter** | **N** | **%** |
| School prestige | 1725 | 45.9 |
| The quality of teaching and teaching staff | 2287 | 60.8 |
| The most convenient to reach | 1172 | 31.2 |
| Advantageous registration fees | 400 | 10.6 |
| Release of foreign university degree | 298 | 7.9 |
| Other | 492 | 13.1 |
| Question 24. Reason to apply in the chosen school | |  |

| **High school attended** | | |
| --- | --- | --- |
| **Parameter** | **N** | **%** |
| Arts studies | 71 | 1.9 |
| Classical studies | 348 | 9.3 |
| Linguistic studies | 282 | 7.5 |
| Scientific studies | 1894 | 50.3 |
| Pedagogical studies | 307 | 8.2 |
| Technical studies | 725 | 19.3 |
| Other | 135 | 3.6 |
| Question 25. High school attended | |  |

| **Fail a class at high school** | | |
| --- | --- | --- |
| **Parameter** | **N** | **%** |
| Yes | 600 | 15.9 |
| No | 3162 | 84.1 |
| Question 27. Fail a class at high school |  |  |

| **High school graduation mark** | | |
| --- | --- | --- |
| **Parameter** | **m (SD)** | |
| Before 1997 | 59.3 (18.4) | |
| After 1997 | 75.9 (11.2) | |
| Question 28. High school graduation mark. Mean (standard deviation) | | |
| \| **Academic title** \| \| \| \| --- \| --- \| --- \| \| **Parameter** \| **N** \| **%** \| \| Bachelor (BSc) \| 2000 \| 53.2 \| \| Master's (MSc) \| 349 \| 9.3 \| \| PhD (PhD) \| 21 \| 0.6 \| \| None \| 1392 \| 37.0 \| \| Question 29. Academic title \|  \|  \| |  |  |
| \| **Attemps of gain access to a degree course** \| \| \| \| --- \| --- \| --- \| \| **Parameter** \| **N** \| **%** \| \| 0 \| 1033 \| 27.5 \| \| 1 \| 1934 \| 51.4 \| \| 2 \| 647 \| 17.2 \| \| 3 \| 104 \| 2.8 \| \| 4 \| 29 \| 0.8 \| \| 5 \| 15 \| 0.4 \| \| Question 30. Attemps of gain access to a degree course \|  \|  \| |  |  |
| **Degree score (highest academic level reached)** | | |
| **Parameter** | **m (SD)** | |
| Degree score | 103.7 (8.4) | |
|  |  |  |
| Question 31. Degree score. Mean (standard deviation) | | |
| \| **Osteopathy school attended** \| \| \| \| --- \| --- \| --- \| \| **Parameter** \| **N** \| **%** \| \| 1 \| 3571 \| 94.9 \| \| 2 \| 179 \| 4.8 \| \| 3 \| 12 \| 0.3 \| \| Question 32. Osteopathy school attended \| \|  \|  \| **Year of course in the current school of osteopathy** \| \| \| \| --- \| --- \| --- \| \| **Parameter** \| **N** \| **%** \| \| 0 \| 721 \| 19.2 \| \| 1 \| 738 \| 19.6 \| \| 2 \| 632 \| 16.8 \| \| 3 \| 646 \| 17.2 \| \| 4 \| 639 \| 17.0 \| \| 5 \| 386 \| 10.3 \| \| 6 \|  \|  \| \| Question 33. Year of course in the current school of osteopathy \| \| \| |  |  |

| **Missed a school year or fail during osteopathy course** | | |
| --- | --- | --- |
| **Parameter** | **N** | **%** |
| Yes | 122 | 3.2 |
| No | 3640 | 96.8 |
| Question 34. Missed a school year or fail during osteopathy course | | |
|  |  |  |
| **Where to practice the profession of osteopath** | | |
| **Parameter** | **N** | **%** |
| Resident region | 3268 | 86.,9 |
| Somewhere else | 1115 | 29.6 |
| Question 35. Where to practice the profession of osteopath | | |
