# Supplementary material for: Osteopathy students profile in Italy: A cross sectional census
Source: PLoS One. 2021 Feb 24;16(2):e0247405. doi: 10.1371/journal.pone.0247405 (PMC7904159; doi:10.1371/journal.pone.0247405)
Supplement: Attachment — Submitted filename: PONE-D-20-33047_reviewer.pdf [file pone.0247405.s004.pdf]

PLOS ONE

Italian Osteopathy Students Profile: A Cross Sectional Census

--Manuscript Draft--

**Manuscript Number:**	PONE-D-20-33047
**Article Type:**	Research Article
**Full Title:**	Italian Osteopathy Students Profile: A Cross Sectional Census
**Short Title:**	Italian Osteopathy Students Profile
**Corresponding Author:**	Michela Persiani, Ph.D<br>Osce-Osteopathic Spine Center Education<br>Bologna, Bo ITALY
**Keywords:**	Manipulation, Osteopathic; Censuses; students; Education, Graduate; Health Education; Students, Health Occupations; Surveys and Questionnaires
**Abstract:**	Background: The Osteopathy Students Analysis (OSA) aims to profile the Italian students in osteopathy as a target population in terms of sociodemographic characteristics, geographical distribution, health status, and previous and ongoing education specifications.<br>Methods: The OSA used a cross-sectional design. A Web-based survey was distributed to the Italian Osteopathic Education Institutions (OEIs). The OSA survey was composed of items organised into four sections: 1. Sociodemographic characteristics (11 items); 2. Geographical distribution (5 items); 3. Health status (3 items); and 4. Previous and ongoing education specifications (16 items). A descriptive sample population analysis was performed. Dichotomous and categorical variables were presented as frequencies and percentages, and continuous variables were displayed as means and standard deviations. Some variables were analysed using a pentenary distribution.<br>Results: 49 out of the 61 OEIs identified matched the inclusion criteria, and among these, 22 accepted to propose the enrolment of their students into the study. The survey was administered to 4,720 students from all the participant OEIs. A total of 3,762 students responded to the survey, accounting for an estimated response rate of 53.7%. The majority of respondents were males (54%), with an average age of 26.9 ± 6.5 years. Almost the totality of the sample was composed of the European Caucasoid ethnic group (99.1%). Respondents were predominantly born in Italy (97.2%). The majority of the sample reported being in good (49.5%) to excellent (38.6%) health. To date, osteopathy students are almost evenly distributed between the two types of curricula (T1 = 46.6%; T2 = 53.4%).<br>Conclusions: The OSA is the first study that aims to profile Italian osteopathy students as a target population in terms of sociodemographic characteristics, geographical distribution, health status, and previous and ongoing education specifications. Future studies should focus on investigating the correlation between the sociodemographic characteristics of students and their academic performance.
**Order of Authors:**	Giacomo Consorti
	Donatella Bagagiolo*
	Andrea Buscemi
	Luca Cicchitti
	Michela Persiani, Ph.D
	Andrea Bergna
**Additional Information:**	
**Question**	**Response**
**Financial Disclosure**<br><br>Enter a financial disclosure statement that describes the sources of funding for the	The author(s) received no specific funding for this work.<br>NO The funders had no role in study design, data collection and analysis, decision to publish, or preparation of the manuscript.

Powered by Editorial Manager® and ProduXion Manager® from Aries Systems Corporation

work included in this submission. Review the submission guidelines for detailed requirements. View published research articles from *PLOS ONE* for specific examples.<br><br>This statement is required for submission and **will appear in the published article** if the submission is accepted. Please make sure it is accurate.<br><br>**Unfunded studies**<br>Enter: *The author(s) received no specific funding for this work.*<br><br>**Funded studies**<br>Enter a statement with the following details:<br>• Initials of the authors who received each award<br>• Grant numbers awarded to each author<br>• The full name of each funder<br>• URL of each funder website<br>• Did the sponsors or funders play any role in the study design, data collection and analysis, decision to publish, or preparation of the manuscript?<br>• **NO** - Include this sentence at the end of your statement: *The funders had no role in study design, data collection and analysis, decision to publish, or preparation of the manuscript.*<br>• **YES** - Specify the role(s) played.<br><br>* typeset	
**Competing Interests**<br><br>Use the instructions below to enter a competing interest statement for this submission. On behalf of all authors, disclose any competing interests that could be perceived to bias this work—acknowledging all financial support and any other relevant financial or non-financial competing interests.<br><br>This statement **will appear in the published article** if the submission is accepted. Please make sure it is accurate. View published research articles from *PLOS ONE* for specific examples.	NO The authors have declared that no competing interests exist.

**NO authors have competing interests**<br><br>Enter: *The authors have declared that no competing interests exist.*<br><br>**Authors with competing interests**<br><br>Enter competing interest details beginning with this statement:<br><br>*I have read the journal's policy and the authors of this manuscript have the following competing interests: [insert competing interests here]*<br><br>* typeset	
**Ethics Statement**<br><br>Enter an ethics statement for this submission. This statement is required if the study involved:<br><br>• Human participants<br>• Human specimens or tissue<br>• Vertebrate animals or cephalopods<br>• Vertebrate embryos or tissues<br>• Field research<br><br>Write "N/A" if the submission does not require an ethics statement.<br><br>General guidance is provided below. Consult the submission guidelines for detailed instructions. **Make sure that all information entered here is included in the Methods section of the manuscript.**	Univeristy of Catania, Deparment of Educational Sciences: Psychology

**Format for specific study types**<br><br>**Human Subject Research (involving human participants and/or tissue)**<br>• Give the name of the institutional review board or ethics committee that approved the study<br>• Include the approval number and/or a statement indicating approval of this research<br>• Indicate the form of consent obtained (written/oral) or the reason that consent was not obtained (e.g. the data were analyzed anonymously)<br><br>**Animal Research (involving vertebrate animals, embryos or tissues)**<br>• Provide the name of the Institutional Animal Care and Use Committee (IACUC) or other relevant ethics board that reviewed the study protocol, and indicate whether they approved this research or granted a formal waiver of ethical approval<br>• Include an approval number if one was obtained<br>• If the study involved *non-human primates*, add *additional details* about animal welfare and steps taken to ameliorate suffering<br>• If anesthesia, euthanasia, or any kind of animal sacrifice is part of the study, include briefly which substances and/or methods were applied<br><br>**Field Research**<br><br>Include the following details if this study involves the collection of plant, animal, or other materials from a natural setting:<br>• Field permit number<br>• Name of the institution or relevant body that granted permission	
**Data Availability**<br><br>Authors are required to make all data underlying the findings described fully available, without restriction, and from the time of publication. PLOS allows rare exceptions to address legal and ethical concerns. See the PLOS Data Policy and FAQ for detailed information.	Yes - all data are fully available without restriction

A Data Availability Statement describing where the data can be found is required at submission. Your answers to this question constitute the Data Availability Statement and **will be published in the article**, if accepted.<br><br>**Important:** Stating 'data available on request from the author' is not sufficient. If your data are only available upon request, select 'No' for the first question and explain your exceptional situation in the text box.<br><br>Do the authors confirm that all data underlying the findings described in their manuscript are fully available without restriction?	
**Describe where the data may be found in full sentences. If you are copying our sample text, replace any instances of XXX with the appropriate details.**<br><br>• If the data are **held or will be held in a public repository**, include URLs, accession numbers or DOIs. If this information will only be available after acceptance, indicate this by ticking the box below. For example: *All XXX files are available from the XXX database (accession number(s) XXX, XXX.*).<br>• If the data are all contained **within the manuscript and/or Supporting Information files**, enter the following: *All relevant data are within the manuscript and its Supporting Information files.*<br>• If neither of these applies but you are able to provide **details of access elsewhere**, with or without limitations, please do so. For example:<br><br>*Data cannot be shared publicly because of [XXX]. Data are available from the XXX Institutional Data Access / Ethics Committee (contact via XXX) for researchers who meet the criteria for access to confidential data.*<br><br>*The data underlying the results presented in the study are available from (include the name of the third party*	All relevant data are within the manuscript and its Supporting Information files.

and contact information or URL).<br>• This text is appropriate if the data are owned by a third party and authors do not have permission to share the data.<br><br>* typeset	
Additional data availability information:	Tick here if the URLs/accession numbers/DOIs will be available only after acceptance of the manuscript for publication so that we can ensure their inclusion before publication.

Powered by Editorial Manager® and ProduXion Manager® from Aries Systems Corporation

Manuscript

Click here to access/download;Manuscript;_OSA_Manuscript_text-19-10-

Italian Osteopathy Students Profile: A Cross Sectional Census

Consorti Giacomo DO giacomo.consorti@isoi.it[1,2], Bagagiolo Donatella DO donatella.bagagiolo@gmail.com*[3], Buscemi Andrea MSc PT DO andreabuscemi@virgilio.it[4,5], Cicchitti Luca DO cicchittiluca@gmail.com[6], Persiani Michela PhD ricerca@spine-center.it[7], Bergna Andrea BSc PT DO andreabergna@soma-osteopatia.it[8,9]; OSA Group[**]

Affiliations

1 Education Department of Osteopathy, ISO - Istituto Superiore di Osteopatia, 120 Ernesto Breda Street, 20126 Milan, Italy.

2 Clinical-based Human Research Department, Research Division, COME Collaboration, Pescara, Italy

3 SSOI - Research Department, SSOI - Scuola Superiore di Osteopatia Italiana, 12 Gian Lorenzo Bernini Square, 10143 Turin, Italy.

4 CSdOI - Centro Studi di Osteopatia Italiano, Marziano 1 Street, 95124 Catania, Italy.

5 Horus cooperativa sociale, Sac. Giovanni Di Giacomo 12 Street, 97100 Ragusa, Italy.

6 AIOT - Accademia Italiana Osteopatia Tradizionale, 127 Caravaggio Street, 65125 Pescara, Italy.

7 OSCE - Osteopathic Spine Center Education, della Liberazione 5 Street, 40128 Bologna, Italy.

8 SOMA - Istituto Osteopatia Milano, 336 F Sarca Road, 20126 Milan, Italy.

9 AISO - Associazione Italiana Scuole di Osteopatia, 127 Caravaggio Street, 65125 Pescara, Italy.

**** Membership of the OSA group is provided in the Acknowledgments.**

Corresponding author: Persiani Michela Ph.D,
Osce-Osteopathic Spine Center Education, Via della Liberazione 5, 40128 Bologna, Italy.
E-mail: ricerca@spine-center.it

Abstract

Background: The Osteopathy Students Analysis (OSA) aims to profile the Italian students in osteopathy as a target population in terms of sociodemographic characteristics, geographical distribution, health status, and previous and ongoing education specifications.

Methods: The OSA used a cross-sectional design. A Web-based survey was distributed to the Italian Osteopathic Education Institutions (OEIs). The OSA survey was composed of items organised into four sections: 1. Sociodemographic characteristics (11 items); 2. Geographical distribution (5 items); 3. Health status (3 items); 4. Previous and ongoing education specifications (16 items). A descriptive sample population analysis was performed. Dichotomous and categorical variables were presented as frequencies and percentages, and continuous variables were displayed as means and standard deviations. Some variables were analysed using a pentenary distribution.

Results: 49 out of the 61 OEIs identified matched the inclusion criteria, and among these, 22 accepted to propose the enrolment of their students into the study. The survey was administered to 4,720 students from all the participant OEIs. A total of 3,762 students responded to the survey, accounting for an estimated response rate of 53.7%. The majority of respondents were males (54%), with an average age of 26.9 ± 6.5 years. Almost the totality of the sample was composed of the European Caucasoid ethnic group (99.1%). Respondents were predominantly born in Italy (97.2%). The majority of the sample reported being in good (49.5%) to excellent (38.6%) health. To date, osteopathy students are almost evenly distributed between the two types of curricula (T1 = 46.6%; T2 = 53.4%).

Conclusions: The OSA is the first study that aims to profile Italian osteopathy students as a target population in terms of sociodemographic characteristics, geographical distribution, health status, and previous and ongoing education specifications. Future studies should focus

on investigating the correlation between the sociodemographic characteristics of students and their academic performance.

Keywords

Manipulation, Osteopathic; Censuses; Students; Education, Graduate; Health Education; Students, Health Occupations; Surveys and Questionnaires

1. Background

Osteopathy is most widely known as a healthcare approach that offers a system of assessment, diagnosis and management of many medical conditions with a range of ‘hands-on’ manual techniques [1][illegible]ecently, osteopathy has been regulated in many countries, including the US, UK, and Australia [1]. Nine countries in Europe (Finland, France, Iceland, Denmark, Lichtenstein, Malta, Portugal, Switzerland and the UK) have legal regulations concerning osteopathy [2–4]. Thus far, despite the regulation process being officially started in Italy, it has not yet been completed. Hence, the osteopathy courses in Italian universities have not yet been establishe[illegible]herefore, osteopathic education is still being carried on in private osteopathic education institutions (OEIs). Benchmarks for Training in Osteopathy were formally defined by the World Health Organization (WHO) in 2010 [5], as a first attempt to make osteopathic education uniform worldwide.

However, osteopathic curricula may differ depending on legal and regulatory structures around the world. Two professional streams in the field of osteopathy have come out amongst the countries with statutory regulation: ‘osteopathic physicians’ and ‘osteopaths’ [1,6].

Osteopathic physicians (practising osteopathic medicine) are qualified physicians with full medical practice rights and can specialise in any branch of medical care, while osteopaths (practising osteopathy) are primary contact healt[illegible]oviders with nationally-defined practise rights who are not licensed to prescribe medications or perform surgery [1,7].

The more recently published European Committee for Standardization (CEN) Standards of Osteopathic Healthcare Provision EN 16686 [8] recognises two types of curriculum organisation proposed in the Benchmarks for Training in Osteopathy. The Type I (T1) programme is suggested for students without a healthcare background. The T1 programme takes 4,800 hours to complete over a 5-year period, including at least 1,000 hours of supervised clinical osteopathic practise and training. The Type II (T2) programme is for

students who have prior training as healthcare professionals. The T2 programme takes 2,000 hours to complete over a minimum of 5 years, including 1,000 hours of supervised osteopathic clinical practise, but this number might be adapted depending on the range of prior training formats [8]. Nowadays, no entry exam is required for school access, and only the previous scholar curriculum is evaluated for T1 or T2 admission.

Worldwide, there has been a growing trend of osteopathic medical school graduates in recent years. The American Osteopathic Association published an annual snapshot of the growth and demographics of the profession. In the US, there are 114,425 Doctors of Osteopathy (DOs) and almost 31,000 students enrolled in colleges of osteopathic medicine, as reported in the 2018 Report on the Osteopathic Medical Profession. These numbers suggest that, if the upward trend continues, DOs are projected to represent more than 20% of all practising physicians by 2030 [9]. In Italy, there are an estimated range 5,000–10,000 osteopaths, as reported by The Forum for Osteopathic Regulation in Europe (FORE) [3]. Recently, osteopathy has been identified as a health profession in Italy, with Law 3/2018 [10], whereby osteopathic education will move from the private OEIs, as it is now, to the higher education sector under the competence of universitie[illegible]his decision could change the training scenario of future osteopaths, especially with the introduction of the admission test to universities. The purpose of the admission restrictions is to regulate the number of graduates in the specific programmes in accordance with the expected demand for specific qualifications in the labour market.

In recent years, a great effort has been made, in the scientific production, to profile the osteopathic professionals in many countries [11–16]. One study that aimed to profile osteopathic practitioners in countries without statutory regulations in osteopathy was the Benelux study [13]. Subsequently, the Osteopathic Practitioners Estimates and RAtes (OPERA) census was conducted to profile the osteopathic profession across Europe [12]. Previous osteopathy studies have largely examined educational issues, as to the role of training methods in medical and osteopathic curricula, with particular reference to Peer Physical Examination (PPE) [14], to investigate the attitudes of Italian osteopathic students

toward self-directed practise [15] and to assess the student perceived preparedness to practise 1 year after graduation [16].

To the best of our knowledge, there are no studies that take into account the sociodemographic characteristics, geographical distribution, health status, and previous and ongoing education specifications of Italian students in osteopathy. Studying the characteristics of this population represents one of the first steps that may help future research to assess the trend of new professionals licensed yearly. Moreover, data will contribute to the evaluation of the numbers for the limited access to university courses and plan and implement a tailored didactic strategy.

The Osteopathy Students Analysis (OSA) is the first census that aims to profile the Italian students in osteopathy as a target population in terms of sociodemographic characteristics, geographical distribution, health status, and previous and ongoing education specifications.

2. Methods

2.1. Design

The OSA used a cross-sectional design. The study was written in accordance with the SUrvey Reporting GuidelinE (SURGE) [17].

The study protocol was approved by the Ethics Committee of the University of Catania Department of Education Science (29 Dec 2018).

2.2. Population

In this survey, the reference population included students attending the Italian OEIs. In order to identify the Italian OEIs, a search strategy was established through a web search using the following keywords: “osteopathic school", "osteopathic university", "osteopathic academy", "osteopathic institute", "osteopathic education", "osteopathic profession", "osteopathic

course", "osteopathic degree", "osteopathic bachelor of science", “osteopathic master of science", and "osteopathic diploma".

The web search was performed independently by two of the authors in May 2019.

Once retrieved, the Italian OEIs were contacted by a member of the team of researchers via e-mail in order to obtain information on the educational level, when not presented in the web site. In this study, the EN16686 standard criteria were adopted, due to the fact that it sets a benchmark for high-quality clinical practise, education, safety and ethics. These criteria help osteopaths to ensure that they provide high-quality healthcare services [4,5,8].

The Italian OEIs were eligible for the study if they fulfilled at least one of the EN16686 criteria: as many as 4,800 hours of education, as many as 240 European Credit Transfer System (ECTS) with at least 60 ECTS of European Qualifications Framework (EQF) level 7 (EQF level 7 degree). The Italian OEIs that organised only postgraduate courses in osteopathy were excluded. The sample population consisted of Italian OEIs students, both in T1 and T2 training programmes, who were enrolled in the 2019–2020 academic year, in each year of the course to ensure better sample representativeness [18]. Before receiving an online questionnaire survey, the Italian OEIs that met the inclusion criteria signed the consent form in electronic format.

2.3 Survey Questionnaire

The OSA survey was composed of items organised into four sections: 1. Sociodemographic characteristics (11 items); 2. Geographical distribution (5 items); 3. Health status (3 items); and 4. Previous and ongoing education specifications (16 items). A detailed overview of the items and the respective answer categories is provided in Additional file 1. The items were selected through a critical analysis of the relevant literature in order to define the specific items of each section [12,19–21]. Personal background features, such as demographic profile, health status and previous education, are reported as the basic knowledge that contributes to the student's performance and knowledge acquisition evaluation [22]. A preliminary pilot-test was administered to 30 Italian graduate students, with a profile similar to that of the sample

population, to test the comprehensibility, relevance, acceptability, feasibility and completeness of the survey information [23]. When the questionnaire was completed, the interpretation of the items was gathered through an interview with the respondents [24]. The researchers reviewed some of the unclear items to make the survey more comprehensible.

The survey was promoted in the Italian Association Schools of Osteopathy website (www.aiso-associazionescuoleosteopatia.it) and was performed through dedicated online survey software, which was accessible both from mobile devices and computers.

One researcher panel was in charge of collecting and accessing the anonymised dataset. The study respected the anonymity and privacy of data in accordance with the General Data Protection Regulation (GDPR Regulation EU 2016/679) [25]. Answers were anonymised and IP addresses were not visible to the researcher. The system automatically managed the link between study ID, e-mail address, and survey status; therefore, it was not possible to identify the respondents.

Administration

The survey was conducted through the performance of a census from 1 October 2019 to 31 January 2020. Each of the Italian OEIs was responsible for administering the survey to all of their students by e-mail with a link to the survey. Participation in the survey was voluntary and anonymous. Consent to participate was requested before the administration of the survey and was given in electronic format. They had to understand and accept all the information reported in the introduction page of the online survey. In order to promote the survey, students were also encouraged to actively take part in it by the personal involvement of researchers and class representatives.

2.2 Statistical analysis

The sample size was calculated based on information obtained through the web and Italian Association of Schools of Osteopathy, which supported an estimated sample of 7,000 students. Considering a standard deviation of 10%, the number of students was expected to

range from 6,300 to 7,700. As reported in the literature, it is assumed that the response rate ranges from 50% to 60% [12] of those receiving the questionnaire; therefore, the number of students participating in the survey was estimated to range from 3,150 to 4,620.

A descriptive sample population analysis was performed. Dichotomous and categorical variables were presented as frequencies and percentages, and continuous variables were displayed as means and standard deviations. Some variables were analysed using a pentenary distribution [26].

3. Results

The screening individuated 61 OEIs. In all, 49 OEIs matched the inclusion criteria, and among these, 22 accepted to propose the enrolment of their students into the study. The survey was administered to 4,720 students from all the participant OEIs. A total of 3,762 students responded to the survey, accounting for an estimated response rate of 53.7%.

3.1 Sociodemographic

The majority of respondents were males (54%), with an average age of 26.9 ± 6.5 years. Almost the totality of the sample was composed of the European Caucasoid ethnic group (99.1%), and more than half of the respondents declared to be Christian (63.6%) (Table 1). The majority of students declared to be a working student (75.9%), and the most represented profession category among them was “freelance” (35.2%). The scholarship of both parents of the respondents showed similarities, accounting for a majority with a high school diploma (mothers: 45.4%; fathers: 46%). Similarly, the working position of both parents accounted for a majority of permanent job positions (mothers: 56.7%; fathers: 57.2%) (Table 2). A wide portion of the sample declared to engage in sports activities (76.2%).

Socio-demographic characteristics		
**Parameter**	**N**	**%**
Total	3762	100
**Gender**		
Female	1721	45.7
Male	2030	54
Other	11	0.3
**Ethnic Group**		
European	3727	99.1
Arab	6	0.16
Indian	2	0.05
Mulatto	11	0.30
Black	3	0.08
Central Asian	3	0.08
American indian	1	0.03
East Asian	4	0.08
Inuit	2	0.04
Mestizio	3	0.08
**Religion**		
Christian	2391	63.6
Atheist	1012	26.9
Muslim	8	0.2
Jew	4	0.1
Other	347	9.2

Table 1. Socio-demographic characteristics

Family of origin characteristics		
**Parameter**	**N**	**%**
**Mother education level**		
Elementary school	114	3
Secondary school	630	16.8
High school	1709	45.4
University	1255	33.4
PhD	54	1.4
**Father education level**		
Elementary school	165	4.5
Secondary school	851	22.6
High school	1731	46
University	946	25.1
PhD	69	1.8
**Mother occupation**		
Freelance	693	18.5
Fixed-term employee	263	6.9

Long-life employee	2133	56.7
Household	673	17.9
**Father occupation**		
Freelance	1415	37.6
Fixed-term employee	163	4.3
Long-life employee	2153	57.2
Household	31	0.9
**Yearly income**		
0 - 15.000 €	222	5.9
15.001 - 28.000 €	678	18
28.001 - 55.000 €	809	21.5
55.000 - 75.000 €	310	8.2
>75.000 €	213	5.7
Not declared	1530	40.7

Table 2. Family of origin characteristics

3.2 Geographical distribution

Respondents were predominantly born in Italy (97.2%), and every Italian region was represented in the sample, ranging from 10 (Valle d'Aosta) to 966 (Lombardia) participants. Despite the geographical provenience being distributed among all the Italian regions, 39.9% of respondents declared to study abroad. As a result, the regions where the respondents declared to study showed a different distribution (Table 3). Moreover, 86.9% of respondents exposed they will work in their region of origin.

Geographical distribution						
**Region**	**Region of birth**		**Region of residence**		**Region of education**	
	**N**	**%**	**N**	**%**	**N**	**%**
Abruzzo	146	3.81	161	4.32	263	7.06
Basilicata	22	0.59	20	0.54	1	0.03
Calabria	47	1.15	37	0.99	5	0.13
Campania	143	3.74	121	3.25	78	2.09
Emilia	367	9.75	394	10.57	487	13.07
Estero	92	2.46	10	0.27	5	0.13
Friuli	50	1.34	43	1.15	37	0.78
Lazio	724	19.43	777	20.85	921	24.72
Liguria	30	0.81	28	0.75	8	0.21
Lombardia	966	25.93	1015	27.24	1179	31.64
Marche	138	3.70	141	3.78	71	1.80
Molise	16	0.43	18	0.48	5	0.13
Piemonte	218	5.74	228	6.12	200	5.16
Puglia	255	6.74	221	5.93	152	4.08

Sardegna	24	0.64	18	0.48	3	0.08
Sicilia	183	4.81	175	4.70	137	3.57
Toscana	104	2.78	108	2.90	71	1.80
Trentino	17	0.46	17	0.46	1	0.03
Umbria	34	0.81	27	0.72	8	0.11
Valle d'Aosta	10	0.27	12	0.32	2	0.05
Veneto	176	4.61	191	5.13	128	3.33

Table 3. Geographical distribution

3.3 Health status

The majority of the sample reported being in good (49.5%) to excellent (38.6%) health. However, 7.1% of the respondents declared that they suffered from musculoskeletal disorders. Only 14 respondents (0.4%) had a severe condition that allowed them to access a disability pension (Table 4).

**Health status**		
**Parameter**	**N**	**%**
**Health status perception**		
Very poor	7	0.2
Poor	45	1.3
Fair	392	10.4
Good	1864	49.5
Excellent	1454	38.6
**Impaired system**		
Central Nervous System	40	1.1
Peripheral Nervous System	21	0.6
Sensorial System	22	0.6
Musculoskeletal System	267	7.1
Cardiovascular System	61	1.6
Endocrine System	139	3.7
Respiratory System	88	2.3
No one	2996	79.6
Other	244	6.5

Table 4. Health status

3.4 Previous and ongoing education

Osteopathy student respondents chose the osteopathy course mostly for the professionalising nature of osteopathic courses in Italy (63.2%) and for the interest in the study topics (60.6%). The choice to engage in the osteopathic school was autonomous for 82.7% of the

respondents, and the driving factor for the school choice was mostly the quality of the teachers (60.8%). To date, osteopathy students are almost evenly distributed between the two types of curricula (T1 = 46.6%; T2 = 53.4%). The most represented high school background for the respondents was the scientific high school (50.3%). Although 15.9% of the sample declared to have failed at least once an academic year during high-school, the graduation grades of the sample were distributed among the 1st and the 2nd pentiles [26]**.** More than half of the sample had a higher education degree. Namely, 53.2% had a BSc, 9.3% had a MSc and only 0.6% already had a PhD. Despite the fact that 36.9% of respondents did not have an academic degree, only 27.5% of them had never attended a university. The average graduation grade for respondents with a higher education degree fell into the 1st pentile (103.7/110 ± 8.4). To date, only a few of the osteopathy students who answered the questionnaire declared to have decided to relocate to another osteopathic school once (4.8%) or twice (0.3%). The distribution of the students among the years of the curriculum was similar among the years (16.8% to 19.6%), apart from the 6th year (10.3%). The year fail rate among the entire curriculum was 3.2% (Table 5).

**Education**		
**Parameter**	**N**	**%**
**Curriculum**		
T1	1754	46.6
T2	2008	53.4
**Academic title**		
BSc	2000	53.2
MSc	349	9.3
PhD	21	0.6
No one	1392	36.9
**Actual academic year**		
1	721	19.2
2	738	19.5
3	632	16.8
4	646	17.2
5	639	17
6	386	10.3

Table 5. Education

4. Discussion

The OSA survey research provides the first large-scale nationally-representative analyses of the sociodemographic characteristics, geographical distribution, health status, and previous and ongoing education specifications of Italian students in osteopathy. Our sample was reasonably representative of Italian osteopathy students, as we had a 53.7% response rate and the participating OEIs were distributed throughout Italy. Data could be considered sufficient to generalize a number of interesting findings concerning the profile of Italian students in osteopathy.

Our results showed a gender distribution comparable with the OPERA-IT study [12]. In fact, the OPERA-IT study showed that, in the newer generation of osteopaths, the female/male ratio tends to be even. The osteopathic profession in Italy has undergone progressive feminisation since its inception [12]. This data seems to be more evident in a European study [16] in which the study sample was predominantly composed of female students (73.8%). Despite the fact that the authors declared that the selected sample might have not been representative of the population of European osteopathy students, it might be speculated that, if the same trend applied to other countries in which the osteopathic profession has been established prior to than in Italy (e.g., the UK), the observed ratio might be plausible. A recent study by Ratti *et al.* [27] showed that the gender variables were not taken into account by patients when they had to choose their osteopaths. These results support the need of an even gender distribution among osteopaths, since patients did not declare to need one gender in particular. Therefore, our findings seem to be in line both with the national trend and needs.

With respect to religious creed, the vast majority of the participant students (73.1%) claimed to be religious and 26.9% to be atheists. These data are in line with those collected last year for the new generation of Italians between 15 and 34 years of age, which would seem to be less religious than the previous ones: Catholics just over 50% and atheists/agnostics over 22%, with Catholics increasing with age to reach a peak of 76.9% among those over 50 [28].

Caring for others can be one of the fundamental aptitudes for the care professions and therefore also for osteopathy. In fact, a physician's skills include patient-centred care, promoting well-being, responding to the needs of patients and society, and sensitivity to the

patient's culture [29]. In particular, osteopathy supports "whole-person care" and the "holistic approach of healthcare", considering the body-mind-spirit paradigm as a fundamental principle of its treatment proposal [30]. The mind-body-spirit medicines combine characteristics of ancient traditional healing systems with the modern biomedical model to create an integrated approach to healthcare [31]. The Christian community provides an alternative nurturing context that permits the full development of the healing relationship and its essential elements: the fact of illness, the profession of healing, and the act of medicine [32]. For example, nursing students state they chose the profession due to altruistic motives of caring for others, and it would seem that Christian students especially have this perspective [33]. Since an educational foundation on spiritual care is integral for the healthcare professions [34,35], the religious characteristics of Italian culture, also present in undergraduate programmes, could favour the choice of care professions. The same students of some healthcare professions, such as nursing sciences, affirm the importance of spiritual awareness in order to address the spiritual needs of patients, incorporating spirituality into education and practice: "Without spirituality, a person is not considered whole" and "Spirituality is an important aspect of a human being" [36–38]. Therefore, spirituality could favour the acquisition of useful skills in the care professions and seems to not negative[illegible]nfluence the acceptance of the Italian schools of osteopathy in the use of teaching methods often used, such as the Peer Physical Examination (PPE) [14], where the students act as models for each other in learning skills in physical examination. Moreover, it has also been claimed that students will be more sensitive and humane and have greater empathy for their patients with this personal experience using PPE [39].

The survey showed that students chose to study osteopathy independently (82.7%). But, in the absence of a previous academic qualification (36.9%), the choice is secondary to a first test of access to a university education (9.4%). However, osteopathic training is sought after a previous academic title. In fact, more than half of the students inte[illegible]wed already had a degree. Training in osteopathy is not considered after a PhD (0.65%), which is probably due to the absence of an academic career. Currently, in Italy, the osteopathic profession gives the

possibility of working only as a freelanc[illegible]5% of students declare to be a freelancer, and this could be justified by a request in specific training in manual therapy to increase work performance [40] and by the demand of patients who increasingly resort to non-pharmacological therapies [41]. Osteopathy has a large scope, from premature newborns [42, 43] to th[illegible]egnant woman [44] to the elderly [45], and is widely used in sports both to improve performance [46] and to prevent and reduce injuries [47–49]. It ranks as the pri[illegible]y medicine for the correction of somatic dysfunction for skel[illegible]muscle pain [44, 50] (visceral [51, 52] and integrative alongside the official medicine for diseases [45, 53, 54]), improving the quality of life [51, 55] and reducing the use of drugs [56], hospitalization times and mortality [57]. The multidisciplinary nature in which osteopathy can be applied makes the osteopath[illegible]fessional a versatile figure with wide working prospects both alone and in ta[illegible]n. The current structure of training in Italy (T1 and T2) allows a comparison in class, mixed classes, between non-working students, workers and teachers, reducing the level of fatigue and increasing the degree of satisfaction of students [58]. It could also be a good teaching strategy to be deepened with further studies and to reduce stress, anxiety and depression, which are very frequent symptoms among students (depression 51.3%, anxiety 66.9%, stress 53%[illegible]59]. It would be useful to evaluate how many graduates in osteopathy work exclusively with osteopathic med[illegible]e. Similarly, it would be useful to assess whether osteopaths without previous healthcare qualifications (T1) decided to acquire an academic qualification after the osteopathic curriculum. This would allow us to understand how osteopathy is placed in the Italian working and cultural system.

Concerning the relationship between students educational choice and education level of their parents, the findings of this study are in accordance with national trends [60]. Most Italian students in osteopathy have parents with a high school diploma (46%), followed by a university degree (29%). Moreover, nearly 60% of the respondents have both parents with long-life employment, with an annual income of € 18,000 and € 55,000, which is also in line with national standards [60]. Considering that the cultural, occupational and social backgrounds of the parents has a decisive influence on the educational choice of the respective grown children

[61, 62], it might be possible to hypothesise that the osteopathic profession is considered a consistent profession with a high standard of living.

Interpreting the geographical distribution through the European nomenclature of territorial units for statistics [63], it is possible to compare the provenience of our participants and the geographical distribution of professional osteopaths presented in the OPERA-IT study [12]. Our data support the geographical distribution observed in the OPERA-IT study among professional osteopaths. Indeed, the North-West macroregion was the most common area of provenience (n = 12,24), followed by the Centre (n = 1,146), North-East (n = 610), South (n = 483) and Islands (n = 207) macroregions.

The students in the assessed sample stated that they had good and excellent health status, with a percentage of 88.1% according to data provided by the Health Behaviour in School-Aged Children study conducted in Italy in 2010 [64], in which the percentage of young Italians who considered themselves in good or very good health was above 85%. Moreover, it is in line with a study on university students from 23 European countries, with differences in culture, in which 87.7% and 90.4% of students of Central-Eastern and Western European countries, respectively, evaluated their own health to be at least “good” [65]. Based on the WHO definition of health [65], it is necessary to consider not only the physical well-being but also the social and mental well-being in order to have a complete understanding of the health of individuals. For this reason, self-perceived health is closely related to multifactorial well-being. Indeed, life satisfaction has been shown to be a strong predictor of self-perceived health [66] and to be associated with health-promoting behaviours, such as not smoking, physical exercise and limiting fat intake [67]. The self-perceived good health of the Italian students in osteopathy could therefore be an appropriate attitude to the skills of the osteopath, which include promoting well-being and responding to needs of patients [68].

The analysis of Italian osteopathy students is interesting considering the growing interest of high school graduates in the faculties in the health professions [69]. The data of the Italian Ministry of Education, University and Research shows that, for the academic year 2018–2019, the faculties of Medicine and Social Healthcare are among the first choices of those who enrol

at universities (32,000 students), along with those in Economics, Engineering and Science. A trend that could be anything but casual and that would be correlated to the labour market. Those who graduate in these subjects, in fact, work and earn more than the other graduates 5 years after obtaining the title, as reported in the latest surveys by the AlmaLaurea Inter-University Consortium (employment rate = 89.3%, with an average net salary 5 years after graduation of 1503.00 euros, data relating to graduates in 2013).

The number of osteopathic medical stud[illegible]s remains among the fastest growing healthcare education choices worldwide and shows no signs of slowing down. The American Association of Colleges of Osteopathic Medicine (AACOM) reported 21,584 osteopathic medical school applications in 2019 (+ 5.8% applications compared to 2018) [70]. Similar growth has been registered by the Australian osteopathic workforce, with 2,627 osteopaths in 2019, compared to 1,601 registered practising osteopaths in 2012 [71]. In the UK, there is a similar trend in terms of numbers of stude[illegible] The General Osteopathic Council published, on 1 March 2019, that there were 5,341 osteopaths on the UK Statutory Register of Osteopaths [72]. Although in the US, Australia and the UK, the majority of students are represented by females (the US 11,347, Australia 1,422, and the UK 2,710) [71–74]. In Italy, the male population is still the most represented (2,030), as reported in the present work. However, albeit slowly, this trend is aligning with other countries. Osteopathic students are younger in the US and Australia, with a mean age of 24 years and 25 years, respectively, compare to in the UK (mean range 31–50 years) and Italy (mean age 29 years) [70–73]. This civil registry difference is given by a differentially designed educational system between countries. So far, only the AACOM has published an exhaustive report on student characteristics. They showed that, although the percentage of under-represented minorities increased by 0.6% (from 16.4% in 2018 to 17% in 2019), Whites and Asians continue to make up more than 75% of the applicant pool. The majority of US applicants have obtained a baccalaureate education degree (life sciences 74.3%, social sciences 9.9%, physical sciences 6.2%, and arts and humanities 5.1%), although most of them came from economically disadvantaged households and were living in a large town [70]. On the other hand, in Italy, under-represented minorities are not

represented, as reported in th[illegible]aper. Almost the totality of the sample was composed of the European Caucasoid ethnic group (3,727). As is such for US students, the majority of Italian osteopathic applicants have obtained baccalaureate education degrees, although most of them came from families with low to middle income bracket households and were living in a large town. The worldwide picture of osteopathic students is almost uniform, suggesting that more information is needed to better understand the future trend of this population in order to assist healthcare practitioners and policy-makers in making decisions concerning consultation and/or referral to osteopat[illegible]

4.1 Lim[illegible]

Our survey research has certain limitations. Firstly, the web-based search strategy to detect the Italian OEIs may have missed some of the institutions that could have participated in the survey, and this could underestimate the population size.

The response rate reached in the survey was considered sufficient. However, some literature suggested that the response rate should be 70–80% [74] for a more confident generalisation of the findings to the entire population. The lower the response rate is, the greater the probability that those who answered would be self-selected rather than randomly chosen, since it is not always possible to determine why other subjects did not respond. Another problem is the potential for nonresponse bias, the probability of which decreases as response rate increases [75].

In the literature, it is widely demonstrated how the occupational field of parents affects the child’s choice of educational fields [76]; moreover, choosing the same educational field as one’s parents helps the grown sons to reach a similar social and occupational status [61]. For these reasons, one of the limitations of this study is the lack of information related to the occupational domain of the parents.

5. Conclusion

The OSA is the first study that aims to profile Italian osteopathy students as a target population in terms of sociodemographic characteristics, geographical distribution, health status, and previous and ongoing education specifications. Our findings have been built upon an estimated representative sample. The outlined profile shows that Italian osteopathy students are evenly distributed between gender, mostly belonging to European Caucasoid ethnic group, and religion, mostly Christians. The educational levels and occupations of the parents of students are comparable, accounting for a high school diploma and long-life employee, respectively. The North-West macroregion accounts for the highest number of students followed by the Centre, North-East, South and Islands macroregions. Overall, students declared to have a good health status with the musculoskeletal system appearing to be the most impaired system. The majority of students are engaged in a T2 curriculum.

Future studies should focus on investigating the correlation between the sociodemographic characteristics of students and their academic performance.

List of abbreviations

Association of Colleges of Osteopathic Medicine (AACOM)

European Committee for Standardization (CEN)

European Credit Transfer System (ECTS)

European Qualifications Framework (EQF)

Forum for Osteopathic Regulation in Europe (FORE)

Osteopathic Education Institutions (OEIs)

osteopathic physicians (DOs)

Osteopathic Practitioners Estimates and RAtes (OPERA)

Osteopathy Students Analysis (OSA)

Peer Physical Examination (PPE)

SUrvey Reporting GuidelinE (SURGE)

Type I (T1)

Type II (T2)

World Health Organization (WHO)

Declarations

Competing interests

All authors have participated in conception and design, or analysis and interpretation of the data; drafting the article or revising it critically for important intellectual content; and approval of the final version.

This manuscript has not been submitted to, nor is under review at, another journal or other publishing venue.

The authors have no affiliation with any organization with a direct or indirect financial interest in the subject matter discussed in the manuscript.

Acknowledgments

The authors thank OSA Group which is composed by: Marasco Marcello (AbeOS), Esposito Nunzia (AEMO), Polistina Stefano (AIFROMM), Giardino Marco, Maggiani Alberto, Scognamiglio Roberto, Zucchi Ferdinando (AIMO), Pizzolorusso Gianfranco (AIOT Marche), Cicchitti Luca (AIOT Pescara), Adragna Vito (AISERCO), Pizzolorusso Gianfranco (ATSAI), Petracca Marco (CERDO), Graffitti Andrea, Tuoto Carmine (Chinesis IFOP), Lombardi Luca (CIO Collegio Italiano Osteopatia), Buscemi Andrea (CSdOI), Adragna Vito (CSOT), Tedesco Giulio (ICOMM), Castagna Carmine, Pisa Viviana, Uberti Stefano (ISO), Chiggiato Emanuele (FULCRO), Pagliaro Roberto (Osteopathic College Trieste), Persiani Michela (OSCE Bologna), Simonelli Marta (SOFI), Bergna Andrea (SOMA Istituto Osteopatia Milano), Bagagiolo Donatella (SSOI Scuola Superiore di Osteopatia Italiana Torino), Tornatore Gianpaolo (TCIO). They also thank Coco Marinella (Department of Biomedical and Biotechnological Sciences, University of Catania, Catania, Italy).

References

1. Adams J, Sibbritt D, Steel A, Peng W. A workforce survey of Australian osteopathy: analysis of a nationally-representative sample of osteopaths from the Osteopathy Research and Innovation Network (ORION) project. BMC Health Serv Res. 2018;18:352.

2. Porthun J, Manschel J. Characteristics, Opportunities, and Challenges of Osteopathy (COCO) in the Perceptions of Osteopaths in Germany, Austria, and Switzerland: Protocol for a Comprehensive Mixed Methods Study. JMIR Res Protoc. 2019;8:e15399.

3. European Federation & Forum for Osteopathy (EFFO). https://www.effo.eu/regulation-in-europe/. Accessed 4 May 2020.

4. Osteopathic Development Group (ODG). https://www.osteointernational.uk/osteopathy-worldwide/. Accessed 4 May 2020.

5. Geneva: WHO,. World Health Organization. Benchmarks for training in osteopathy. 2010.

6. Osteopathic International Alliance. Osteopathy and osteopathic medicine: a global view of practice, patients, education and the contribution to healthcare delivery. 2013. http://oialliance.org/resources/oia-status-report/. Accessed 13 May 2020.

7. American Association of Colleges of Osteopathic Medicine. The difference between U.S.-trained osteopathic physicians and osteopaths trained abroad. 2017. http://www.aacom.org/become-a-doctor/about-om/US-vs-abroad. Accessed 4 May 2020.

8. CEN//TC. Project Committee. 2015 Services in Osteopathy. Osteopathic healthcare.

9. American Osteopathic Association Report on the Osteopathic Medical Profession. 2018. https://osteopathic.org/about/aoa-annual-report/. Accessed 4 May 2020.

10. Law 3/2018,. 2018.

11. Vaucher P, Macdonald RJD, Carnes D. The role of osteopathy in the Swiss primary health care system: a practice review. BMJ Open. 2018;8:e023770.

12. Cerritelli F, van Dun PLS, Esteves JE, Consorti G, Sciomachen P, Lacorte E, et al. The Italian Osteopathic Practitioners Estimates and RAtes (OPERA) study: A cross sectional

survey. PLOS ONE. 2019;14:e0211353.
13. van Dun PLS, Nicolaie MA, Van Messem A. State of affairs of osteopathy in the Benelux: Benelux Osteosurvey 2013. Int J Osteopath Med. 2016;20:3–17.
14. Consorti F, Mancuso R, Piccolo A, Consorti G, Zurlo J. Evaluation of the acceptability of Peer Physical Examination (PPE) in medical and osteopathic students: a cross sectional survey. BMC Med Educ. 2013;13:111.
15. D'Alessandro G, Consorti G, Cerritelli F. Self-directed learning and practice of Italian osteopathic students during summer break: a cross-sectional survey. BMC Complement Altern Med. 2019;19:201.
16. Luciani E, Consorti G, van Dun PLS, Merdy O, Lunghi C, Petracca M, et al. An overview of osteopathy graduates' perceived preparedness at transition from educational environment to clinic environment one year after graduation: a cross sectional study. BMC Med Educ. 2018;18:319.
17. Bennett C, Khangura S, Brehaut JC, Graham ID, Moher D, Potter BK, et al. Reporting Guidelines for Survey Research: An Analysis of Published Guidance and Reporting Practices. PLoS Med. 2011;8:e1001069.
18. Fincham JE. Response rates and responsiveness for surveys, standards, and the Journal. Am J Pharm Educ. 2008;72:43.
19. Abbiati M, Baroffio A, Gerbase MW. Personal profile of medical students selected through a knowledge-based exam only: are we missing suitable students? Med Educ Online. 2016;21:29705.
20. Oliveira ACP de, Machado APG, Aranha RN. Identification of factors associated with resilience in medical students through a cross-sectional census. BMJ Open. 2017;7:e017189.
21. Finucane P, Flannery D, McGrath D, Saunders J. Demographic attributes and knowledge acquisition among graduate-entry medical students. Med Teach. 2013;35:134–8.
22. Salem RO, Al-Mously N, Nabil NM, Al-Zalabani AH, Al-Dhawi AF, Al-Hamdan N. Academic and socio-demographic factors influencing students' performance in a new Saudi

medical school. Med Teach. 2013;35:S83–9.

23. de Vet HCW, Terwee CB, Mokkink LB, Knol DL. Measurement in Medicine: A Practical Guide. Cambridge: Cambridge University Press; 2011. doi:10.1017/CBO9780511996214.

24. Draugalis JR, Coons SJ, Plaza CM. Best Practices for Survey Research Reports: A Synopsis for Authors and Reviewers. Am J Pharm Educ. 2008;72:11.

25. General Data Protection Regulation (GDPR). (EU 2016/679). https://gdpr-info.eu. Accessed 20 May 2020.

26. Vertecchi B, Agrusti G. Laboratorio di valutazione. Roma; Bari: Laterza; 2008.

27. Ratti S, Zecchillo D, Acquati A, Acquino A, Uberti S, Pisa V,. Il genere dell'osteopata condiziona le preferenze del paziente? Uno studio cross-sectional. In: Proceeding of the 4th Congress of Registro degli Osteopati d'Italia. 2018. https://www.registro-osteopati-italia.com/wp-content/uploads/2018/06/Ratti-Silvia_Il-genere-dell%E2%80%99osteopata_POSTER.pdf. Accessed 9 Jul 2020.

28. Sondaggio Doxa su religiosità e ateismo. https://www.uaar.it/doxa2019/. Accessed 7 Jul 2020.

29. Competencies for the physician assistant profession. JAAPA. 2005;18(7):16-18. http://www.jaapa. com/issues/j20050701/articles/comp0705.htm. Accessed September 18, 2006.

30. Zegarra-Parodi R, Draper-Rodi J, Haxton J, Cerritelli F. The Native American heritage of the body-mind-spirit paradigm in osteopathic principles and practices. Int J Osteopath Med. 2019;33–34:31–7.

31. Lloyd LF, Dunn LR. Mind-body-spirit medicine: Interventions and resources: J Am Acad Physician Assist. 2007;20:31–5.

32. Toombs SK. The healing relationship: Edmund Pellegrino's philosophy of the physician-patient encounter. Theor Med Bioeth. 2019;40:217–29.

33. Johanson LS. Six Principles for Remaining Positive in a Negative Work Environment. J Christ Nurs Q Publ Nurses Christ Fellowsh. 2019;36:E21–4.

34. Boswell C, Cannon SB, Miller J. Students' Perceptions of Holistic Nursing Care. Nurs

Educ Perspect. 2013;34:329–33.

35. Cerra A, Fitzpatrick JJ. CAN IN-SERVICE Education HELP Prepare Nurses for Spiritual Care?: J Christ Nurs. 2008;25:204–9.

36. Narayanasamy A. The impact of empirical studies of spirituality and culture on nurse education. J Clin Nurs. 2006;15:840–51.

37. Tiew LH, Creedy DK, Chan MF. Student nurses' perspectives of spirituality and spiritual care. Nurse Educ Today. 2013;33:574–9.

38. Hovland C, Niederriter J, Thoman J. Spirituality and Interprofessional Healthcare Education: An Exploratory Study. J Christ Nurs Q Publ Nurses Christ Fellowsh. 2018;35:E47–52.

39. Outram S, Nair BR. Peer physical examination: time to revisit? Med J Aust. 2008;189:274–6.

40. Hearle D, Lawson S. Continuing Professional Development Engagement—A UK-based Concept Analysis: J Contin Educ Health Prof. 2019;39:251–9.

41. Complementary, Alternative, or Integrative Health: What's In a Name? 2016. https://nccih.nih.gov/health/integrative-health. Accessed 19 Jun 2020.

42. Lanaro D, Ruffini N, Manzotti A, Lista G. Osteopathic manipulative treatment showed reduction of length of stay and costs in preterm infants: A systematic review and meta-analysis. Medicine (Baltimore). 2017;96:e6408.

43. Cerritelli F, Pizzolorusso G, Renzetti C, Cozzolino V, D'Orazio M, Lupacchini M, et al. A Multicenter, Randomized, Controlled Trial of Osteopathic Manipulative Treatment on Preterms. PLOS ONE. 2015;10:e0127370.

44. Franke H, Franke J-D, Belz S, Fryer G. Osteopathic manipulative treatment for low back and pelvic girdle pain during and after pregnancy: A systematic review and meta-analysis. J Bodyw Mov Ther. 2017;21:752–62.

45. Buscemi A, Pennisi V, Rapisarda A, Pennisi A, Coco M. Efficacy of osteopathic treatment in patients with stable moderate-to-severe chronic obstructive pulmonary disease: a randomized controlled pilot study. J Complement Integr Med. 2019;17. doi:10.1515/jcim-

2018-0128.
46. Buscemi A, Petralia M, Ramaci T, Rapisarda A, Provazza C, Di Corrado D, et al. Ergojump evaluation of the explosive strength in volleyball athletes pre‑ and post‑ fascial treatment. Exp Ther Med. 2019. doi:10.3892/etm.2019.7628.
47. Valentina Perciavalle, Fulvia Fichera, Carmelo Crocellà, Emiliano Raciti, Giuseppe Privitera, Vincenzo Audino, Alessandro Rapisarda, Maria Cristina Petralia, Marinella Coco, Andrea Buscemi. L'infortunio nel rugby: ipotesi di intervento. L'infortunio Nel Rugby Ipotesi Interv. 2014;Movimento, 30:1–4.
48. Brumm L, Janiski C, Balawender J, Feinstein A. Preventive Osteopathic Manipulative Treatment and Stress Fracture Incidence Among Collegiate Cross-Country Athletes. J Am Osteopath Assoc. 2013;113:882–90.
49. Ofei-Dodoo S, Black JL, Kirkover MA, Lisenby CB, Porter AST, Cleland PM. Collegiate Athletes' Perceptions of Osteopathic Manipulative Treatment. Kans J Med. 2020;13:147–51.
50. Franke H, Franke J-D, Fryer G. Osteopathic manipulative treatment for nonspecific low back pain: a systematic review and meta-analysis. BMC Musculoskelet Disord. 2014;15:286.
51. Tamer S, Öz M, Ülger Ö. The effect of visceral osteopathic manual therapy applications on pain, quality of life and function in patients with chronic nonspecific low back pain. J Back Musculoskelet Rehabil. 2017;30:419–25.
52. Lagrange A, Decoux D, Briot N, Hennequin A, Coudert B, Desmoulins I, et al. Visceral osteopathic manipulative treatment reduces patient reported digestive toxicities induced by adjuvant chemotherapy in breast cancer: A randomized controlled clinical study. Eur J Obstet Gynecol Reprod Biol. 2019;241:49–55.
53. Wells RE, Baute V, Wahbeh H. Complementary and Integrative Medicine for Neurologic Conditions. Med Clin North Am. 2017;101:881–93.
54. Alessandro Rapisarda e Andrea Buscemi AR e AB. Effects of osteopathy on haemochrome and blood pressure. International Journal of Education and Research. Eff Osteopat Haemochrome Blood Press. 2015;Vol. 3 No. 1:421–30.
55. Buscemi A, Martino S, Scirè Campisi S, Rapisarda A, Coco M. Endocannabinoids

release after Osteopathic Manipulative Treatment. A brief review. J Complement Integr Med. 2020.

56. Sousa IMC de, Hortale VA, Bodstein RC de A. Medicina Tradicional Complementar e Integrativa: desafios para construir um modelo de avaliação do cuidado. Ciênc Saúde Coletiva. 2018;23:3403–12.

57. Noll DR, Degenhardt BF, Johnson JC. Multicenter Osteopathic Pneumonia Study in the Elderly: Subgroup Analysis on Hospital Length of Stay, Ventilator-Dependent Respiratory Failure Rate, and In-hospital Mortality Rate. J Am Osteopath Assoc. 2016;116:574.

58. Kutyło Ł, Łaska-Formejster A, Ober-Domagalska B. Fatigue among working and non-working students: a sociological analysis of the environmental determinants of its level. Med Pr. 2019. doi:10.13075/mp.5893.00851.

59. Iqbal S, Gupta S, Venkatarao E. Stress, anxiety and depression among medical undergraduate students and their socio-demographic correlates. Indian J Med Res. 2015;141:354–7.

60. Studenti e Bacini Universitari. https://www.istat.it/it/files/2016/11/Studenti-e-bacini-universitari.pdf. Accessed 13 Jun 2020.

61. Hodkinson P, Sparkes AC. Careership: a sociological theory of career decision making. Br J Sociol Educ. 1997;18:29–44.

62. Jonsson JO, Grusky DB, Di Carlo M, Pollak R, Brinton MC. Microclass Mobility: Social Reproduction in Four Countries. Am J Sociol. 2009;114:977–1036.

63. Europäische Kommission, editor. Regions in the European Union: nomenclature of territorial units for statistics ; NUTS 2013/EU-28. Luxembourg: Publ. of the Europ. Union; 2015.

64. Cavallo F, Giacchi M, Vieno A, Galeone D, Tomba A, Lamberti A, Nardone P, Andreozzi S (Ed.). Studio HBSC in Italia (Health Behavior in School-aged children): rapporto sui dati 2010. Roma: Istituto Superiore di Sanità; 2010. (Rapporti ISTISAN, 13/5). Available from: www.iss.it/ publ/index.php?lang=1&id=2685&tipo=5.

65. WHO definition of health. https://www.who.int/about/who-we-are/constitution. Accessed

13 Jun 2020.
66. Al-Windi A. The relations between symptoms, somatic and psychiatric conditions, life satisfaction and perceived health. A primary care based study. Health Qual Life Outcomes. 2005;3:28.
67. Grant N, Wardle J, Steptoe A. The Relationship Between Life Satisfaction and Health Behavior: A Cross-cultural Analysis of Young Adults. Int J Behav Med. 2009;16:259–68.
68. Sciomachen P, Arienti C, Bergna A, Castagna C, Consorti G, Lotti A, et al. Core competencies in osteopathy: Italian register of osteopaths proposal. Int J Osteopath Med. 2018;27:1–5.
69. Interesse dei giovani nei confronti della professione. https://www.agi.it/cronaca/facolta_preferite_universitari_italia-6132110/news/2019-09-04/. Accessed 13 Jun 2020.
70. Osteopathic Medical School Application, completed by applicants. https://www.aacom.org/reports-programs-initiatives/aacom-reports#Latest. Accessed 13 Jun 2020.
71. Osteopathy Board of Australia Registrant data. 2020. https://www.osteopathyboard.gov.au/About/Statistics.aspx. Accessed 13 Jun 2020.
72. General Osteopathic Council Annual Report and Accounts 2018-19. www. org.uk/annualreport. Accessed 13 Jun 2020.
73. The 2019 Osteopathic Medical Profession (OMP) Report. https://osteopathic.org/wp-content/uploads/OMP2019-Report_Web_FINAL.pdf. Accessed 13 Jun 2020.
74. Draugalis JR, Plaza CM. Best Practices for Survey Research Reports Revisited: Implications of Target Population, Probability Sampling, and Response Rate. Am J Pharm Educ. 2009;73:142.
75. Guo Y, Kopec JA, Cibere J, Li LC, Goldsmith CH. Population Survey Features and Response Rates: A Randomized Experiment. Am J Public Health. 2016;106:1422–6.
76. van de Werfhorst HG, Luijkx R. Educational Field of Study and Social Mobility: Disaggregating Social Origin and Education. Sociology. 2010;44:695–715.

Click here to access/download
Supporting Information
Additional_file_1.docx
